# Metagenomic Reveals the Role of Autochthonous *Debaryomyces hansenii* in the Fermentation and Flavor Formation of Dry Sausage

**DOI:** 10.3390/foods14010140

**Published:** 2025-01-06

**Authors:** Qian Chen, Siyuan He, Mengtong Li, Yumeng Sui, Baohua Kong, Rongxin Wen

**Affiliations:** 1 College of Food Science, Northeast Agricultural University, Harbin 150030, China; chenqianego7@126.com (Q.C.); hesiyuan0068@163.com (S.H.); limengtong092320@163.com (M.L.); suiyumenglz@126.com (Y.S.); kongbh@163.com (B.K.); 2 Yantai Key Laboratory of Characteristic Agricultural Bioresource Conservation & Germplasm Innovative Utilization, School of Life Sciences, Yantai University, Yantai 264005, China

**Keywords:** dry sausage, volatile compound, *Debaryomyces hansenii*, metagenomic, microbial community, metabolic pathway

## Abstract

The effect of *Debaryomyces hansenii* SH4, a typical aroma enhancer, on flavor formation of the dry fermented sausage was investigated using gas chromatography-mass spectrometry and metagenomic sequencing. The results showed that inoculation with *D. hansenii* SH4 promoted volatile compound formation from carbohydrate and amino acid metabolism and accelerated ester synthesis. The enzymes, genes, and microorganisms involved in the formation pathway of volatile compounds based on microbial metabolism were predicted and constructed into a metabolic pathway network. *D. hansenii*, *Lactobacillus curvatus*, *Lactobacillus sakei*, *Lactobacillus plantarum*, *Leuconostoc fallax*, *Weissella minor*, and *Staphylococcus* and *Candida* species were found to be the predominant functional microbes for flavor development in dry sausage. This study established a new insight into the metagenome-based bioinformatic effects of *D. hansenii* SH4 as a starter culture on the microbial synthesis of key volatile compounds in dry sausage.

## 1. Introduction

Dry sausage, a classic fermented meat product from China, has been a favorite among consumers for centuries due to its distinct texture and fermented flavor. Traditional dry sausage is produced by spontaneous fermentation. Briefly, raw meat and auxiliary ingredients mixtures are stuffed into casings and spontaneously fermented for around 10 days. During fermentation, dry sausage is regarded as a micro-ecosystem containing various microbiomes that contribute to the breakdown and metabolism of the primary ingredients (proteins, carbohydrates, and lipids). These processes result in the production of volatile compounds that affect the flavor and quality of the finished product [1]. However, the structure of the microbial community is usually altered due to changes in the fermentation environment. Thus, a fermentation process dependent on the natural microbial community results in inconsistent sausage flavor and quality. To maintain the quality and stability of the products, the fermentation process is progressively standardized using starter cultures [2].

Inoculation of starter cultures may produce perturbations in the food system that alter the initial microbiota composition of the raw materials and impact the final products’ flavor and quality [3]. Such changes in flavor might be related to the composition and metabolism of the microbial community [4]. A recent study revealed that *Debaryomyces hansenii* was the dominant species in the fungal community of traditional dry sausages [5]. Building on this finding, several autochthonous strains of *D. hansenii* were isolated from traditional dry sausages, with *D. hansenii* SH4 identified as the most effective flavor producer. When used as a starter culture, *D. hansenii* SH4 significantly enhanced the flavor profile and sensory characteristics of dry sausage [6]. However, the mechanisms behind the effects of this yeast on the composition of the microbial community and the synthesis of flavor compounds in dry sausage are not yet understood.

Metagenomics sequencing techniques allow for exploring the microbial community structures, flavor metabolic pathways, and related genes in fermented products. Metagenomics is a bioinformatics approach that enables the direct construction of almost the entire microbial genomes from sequencing data [7]. This genome-centric approach can be used to analyze the effect of a certain change on the microbial community and the metabolic pathways that are directly related to the quality of the product and thus can be used to optimize a manufacturing process [8]. In this way, metagenomics can be used to evaluate how starter culture affects a product’s microbial diversity.

Therefore, this work aims to elucidate the mechanisms of the effects of *D. hansenii* SH4 inoculation on the flavor profile of dry sausage. The naturally fermented sausage and sausage inoculated with *D. hansenii* SH4 were analyzed using metagenomics and gas chromatography-mass spectrometry. The differences in the volatile compositions and relative abundances of microorganisms and functional genes were compared. In addition, to better understand the flavor formation and metabolic regulatory mechanisms of the microbiota in dry sausage, the formation pathways of volatile compounds and the associated enzymes, coding genes, and microorganisms were analyzed. Finally, the metabolic pathway networks of volatile compounds were predicted and constructed. These findings will contribute to a clearer understanding of the composition and aromatic role of the microbial community in dry sausage. They will also help identify the specific beneficial microorganisms responsible for flavor formation in the sausages.

## 2. Materials and Methods

### 2.1. Yeast Starter Culture Preparation

*D. hansenii* SH4, the autochthonous strain employed in this investigation, was isolated from traditional spontaneous dry sausages [6]. This strain has been identified, and the DNA sequences were submitted to GenBank under accession number OK448347. The stock cultures of *D. hansenii* SH4 were reactivated for 24 h at 28 °C in yeast extract peptone dextrose (YPD) broth. After centrifugation (6000× *g* for 10 min at 4 °C), the cells were collected, and they were twice washed with 0.85% sterile saline. The yeast cells were suspended in sterile saline before use.

### 2.2. Dry Sausage Preparation

To prepare the dry sausages, 400 g of pork back fat and 3600 g of lean pork were minced through a 1.5 cm orifice plate. The minced pork was combined with 40 g of dextrose, 100 g of NaCl, 12 g of monosodium glutamate, 40 g of *Daqu* (traditional Chinese wine), 0.36 g of sodium nitrite, and 32 g of mixed spices (fennel, clove, round cardamom, orange peel, *Amomum villosum*, *Angelica dahurica*, and cassia bark).

Three independent sausage batches were manufactured, and two treatments were prepared for each batch. The control sausage was not inoculated with a starter culture, whereas the Dh-SH4 sausage was inoculated with *D. hansenii* SH4 at a concentration of 10^6^ CFU/g meat. After carefully combining the meat with additional ingredients, it was stuffed into 3.0 cm diameter porcine natural casings. The sausages underwent two rounds of fermentation in an incubator: a one-day fermentation at 25 ± 2 °C and 30–50% relative humidity, and an eleven-day fermentation at 25 ± 2 °C and 75–80% relative humidity. After fermentation, three independent batches (biological replicates) of control sausage and Dh-SH4 sausage were sampled for further study.

### 2.3. Analysis of Physical Properties and Microbial Counts

The moisture content of dry sausages was determined using the techniques provided in the AOAC [9]. The water activity (aw) was determined using an Aqualab Aw meter (Decagon Devices, Pullman, WA, USA). The pH was determined using a pH meter (2018C132-1, Sardonis Scientific Instruments Co., Ltd., New Delhi, India) The yeast counts were determined using YPD agar plates incubated for five days at 28 °C. The lactic acid bacteria (LAB) counts were determined using de-Man Rogosa Sharpe agar plates incubated for 72 h at 30 °C.

### 2.4. Volatile Compound Analysis

Minced sausages (2.0 g) were extracted and analyzed for the volatile compounds using solid-phase microextraction (SPME) and gas chromatography-mass spectrometry (GCMS-QP2020 NX, Shimadzu Co., Kyoto, Japan) in accordance with the technique described by Wen et al. [5]. Compounds were identified by matching the mass spectra from the NIST 17 mass spectra library and the Kovats retention indexes from the literature (standard alkanes of C6–C20 were used). Semi-quantitative data (μg/kg) were produced by dividing the peak areas of each detected component by the peak area of the internal standard (1,2-dichlorobenzene) and multiplying this ratio by the internal standard’s initial concentration.

### 2.5. Metagenomic Analysis

#### 2.5.1. DNA Extraction and Metagenomic Sequencing

The sausages were removed from their casings and crushed into mince. Using the cetyltrimethyl ammonium bromide (CTAB) technique, 0.5 g of the minced sausage was used to extract total genomic DNA. DNA integrity was checked on 1% agarose gel, and concentration was determined using a Qubit^®^ dsDNA Assay Kit with a Qubit^®^ 2.0 Fluorometer (Life Technologies, Carlsbad, CA, USA) [10]. Using an Illumina HiSeq platform (Novogene Bioinformatics Technology, Tianjin, China), the libraries were sequenced.

#### 2.5.2. Bioinformatic Analysis

The raw data were cleaned, blasted, processed, and assembled to produce the gene inventory (unigenes). The unigenes were blasted to the bacteria, archaea, fungi, and virus sequences from the NCBI NR database using DIAMOND software (V0.9.9) [11]. Linear discriminant analysis effect size (LEfSe) comparison analyses were used to find the different species between samples. In addition, the unigenes were blasted to the functional databases (KEGG and eggNOG databases) using DIAMOND software (V0.9.9) [12]. Based on the outcomes of taxonomic assignment and function annotation, the functional genes and enzymes associated with the formation pathway of each volatile compound were obtained. The relative contribution of each microorganism to the volatile compound formation was calculated according to the sum of its annotated functional gene abundance.

### 2.6. Statistical Analysis

All sausage samples had three replicates. For physical analysis, microbial counts, and volatile compound analysis, the data of the results were performed using the General Linear Models procedure of the Statistix 8.1 software package (Analytical Software, St Paul, MN, USA). The data were reported as the mean ± standard error (SE). Variance (ANOVA) analysis with Tukey’s multiple comparisons was applied to assess the significant difference between the treatment effects (*p* < 0.05).

## 3. Results and Discussion

### 3.1. Physical Properties and Microbial Counts

Table A1 shows the moisture content, aw, pH values, yeast, and LAB counts in dry sausages. After fermentation, higher moisture content, aw, and pH values were observed in the Dh-SH4 sausage than in the control sausage (*p* < 0.05), which were similar to the findings of Corral et al. [13], who found that *D. hansenii* inoculation could regulate the release of water during fermentation, leading to sausages with increased aw and moisture content. Additionally, a minor rise in pH after *D. hansenii* inoculation may have resulted from yeasts consuming organic acids [14]. The Dh-SH4 sausage had considerably increased yeast counts compared to the control sausage (*p* < 0.05). Similar findings were seen in other sausages inoculated with yeast strains [15], confirming the successful implantation of the yeast strains inoculated. Interestingly, the Dh-SH4 sausage showed higher LAB counts than the control sausage (*p* < 0.05), suggesting that *D. hansenii* DH4 inoculation promoted the development of lactic acid bacteria in dry sausages. This effect may be attributed to yeast–LAB interactions [16], such as the timely uptake of lactic acid by yeast, which helps maintain a relatively high pH conducive to LAB growth and prevents excessive acidification. Additionally, yeast may contribute to LAB growth by supplying essential nutrients, including free amino acids and vitamins [17].

### 3.2. Volatile Compounds

**Figure 1:**
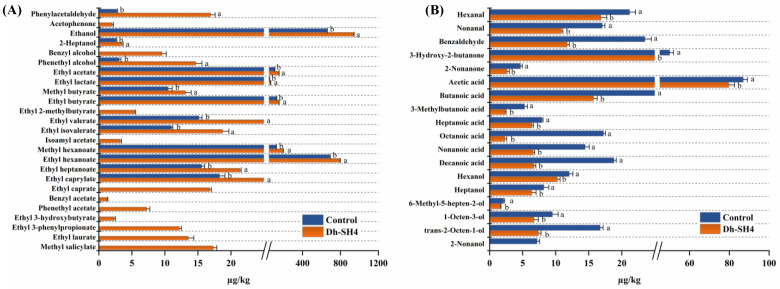
Volatile compound contents in Dh-SH4 and control dry sausages: compounds are significantly higher in Dh-SH4 sausages (**A**), and compounds are significantly higher in control sausages (**B**). ^a,b^ Refers to the significant differences between the dry sausages (*p* < 0.05). Control: natural fermentation; Dh-SH4: inoculation with *D. hansenii* SH4.

The types and contents of the volatile compounds in the naturally fermented sausage (control) and sausage inoculated with *D. hansenii* SH4 were analyzed to investigate the effect of *D. hansenii* SH4 as a starter culture on the formation of the flavor profile. In the control and Dh-SH4 sausages, a total of 70 and 80 volatile compounds were found, respectively (Table A2). The volatile compounds with significant differences in content between the two sausages are presented in Figure 1. The contents of 25 volatile compounds, including phenylacetaldehyde, acetophenone, benzyl alcohol, phenethyl alcohol, 2-heptanol, ethanol, and 19 esters, were significantly higher in the DH-SH4 sausage than in the control sausage (*p* < 0.05) (Figure 1A). These compounds are primarily produced by the amino acid metabolism, carbohydrate metabolism, and ester synthesis of microorganisms [18], suggesting that *D. hansenii* SH4 as a starter culture may result in more active metabolic pathways for the generation of these compounds in dry sausages. By contrast, the contents of 19 volatile compounds, including three aldehydes, two ketones, seven acids, and seven alcohols, were significantly lower in the Dh-SH4 sausage than in the control sausage (*p* < 0.05) (Figure 1B). Fatty acid oxidation and degradation are the primary processes that lead to the synthesis of these molecules’ compounds [19]. These findings suggest that *D. hansenii* SH4 as a starter culture may reduce the metabolic activities involving fatty acids in dry sausages. Previous studies have reported that the composition and metabolic function of the microbial community in dry sausages are closely related to the development of volatile compounds [5,20]. Therefore, the changes in the flavor profile of dry sausage after inoculation with *D. hansenii* SH4 may be largely attributed to the changes in the structure of the microbial community. The identification of the linkages between the metabolic functions of the microbial community and the production of volatile compounds in sausage fermentation systems could potentially improve the flavor of dry sausages.

### 3.3. Microbial Community Diversity

**Figure 2:**
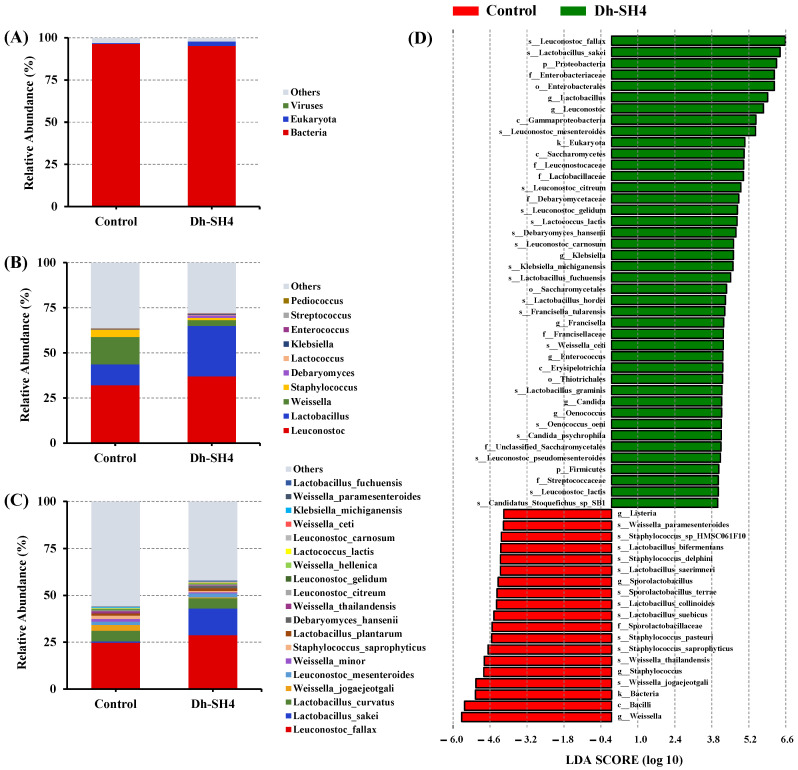
The relative abundance at the kingdom level (**A**), genus level (**B**), and species level (**C**), as well as linear discriminant analysis effect size comparison (**D**) of microbial communities in dry sausages. Control: natural fermentation; Dh-SH4: inoculation with *D. hansenii* SH4.

To investigate the mechanisms of flavor formation in the dry sausage inoculated with *D. hansenii* SH4, the naturally fermented sausage and sausage inoculated with *D. hansenii* SH4 were analyzed using metagenomic sequencing. As shown in Table A3, a total of 38.90 Gbp raw reads were obtained. After strict quality control, 38.72 Gbp remained. The sequencing process demonstrated a high level of quality, as over 95% of the reads had sequencing errors below 1% (Q20) [21]. After gene prediction, 113,182, and 117,594 open reading frames (ORFs) were found in the control and Dh-SH4 sausages, respectively. The structures of the microbial communities in the dry sausages are shown in Figure 2. At the kingdom level (Figure 2A), the microbial community in the dry sausage is dominated by bacteria and fungi. Further analysis at the genus level (Figure 2B) showed that *Leuconostoc*, *Lactobacillus*, *Weissella*, *Staphylococcus*, and *Debaryomyces* predominated in the microbial community, with total relative abundances reaching 62.83% and 70.76% in the control and Dh-SH4 sausages, respectively. The microbial communities experienced a remarkable and immediate change with the inoculation of *D. hansenii* SH4. The relative abundances of *Leuconostoc*, *Lactobacillus*, and *Debaryomyces* were higher in the Dh-SH4 sausage than in the control sausage, whereas those of *Weissella* and *Staphylococcus* were lower. These results indicate that inoculation with *D. hansenii* SH4 changed not only the relative abundance of fungi but also the composition and relative abundance of bacteria.

Further analysis at the species level (Figure 2C) revealed that the Dh-SH4 sausage had a higher relative abundance of *D. hansenii*. These results were in line with the results in Table A1, indicating that the strain successfully colonized the dry sausage as a starter culture. Furthermore, when compared to the control sausage, the relative abundances of several lactic acid bacteria increased in the Dh-SH4 sausage. For example, the relative abundances of *Leuconostoc fallax*, *Lactobacillus sakei*, *Lactobacillus curvatus*, *Leuconostoc mesenteroides*, and *Lactobacillus plantarum* were 24.79%, 0.69%, 5.41%, 1.83%, and 1.12%, respectively, in the control sausage, and increased to 28.79%, 14.22%, 5.67%, 2.15%, and 1.48%, respectively, in the Dh-SH4 sausage. These findings supported those in Table A1, suggesting that the environment in the Dh-SH4 sausage may be better for the development of lactic acid bacteria. Previous studies have reported that lactic acid bacteria are the most prevalent bacteria during the fermentation of dry sausage [20]. Lactic acid bacteria can metabolize carbohydrates to produce organic acids, which gives a sour taste to the product [22]. In addition, it can promote the production of a variety of volatile compounds [23]. Some lactic acid bacteria, such as *L. curvatus*, *L. sake*i, and *L. plantarum*, have been isolated and studied as starter cultures for improving the quality and flavor formation of fermented sausages [24]. By contrast, the relative abundances of some strains were lower in the Dh-SH4 sausage than in the control sausage. For example, the relative abundances of *Weissella jogaejeotgali*, *Weissella minor*, and *Staphylococcus saprophyticus* were 3.09%, 1.39%, and 1.78% in the control sausage, respectively, and decreased to 0.55%, 0.60%, and 0.59% in the Dh-SH4 sausage, respectively. Overall, inoculation with *D. hansenii* SH4 resulted in substantial changes in the microbial community’s composition in dry sausages, which was one of the reasons for the changes in the quality and flavor of the sausages.

LEfSe was used to identify significant differences in the microbial communities of the control and Dh-SH4 sausages. Figure 2D shows the differences in the relative abundances of microorganisms (from the kingdom to the species level) of different sausages. The Dh-SH4 sausage exhibited a significantly higher relative abundance of 42 microorganisms and a lower relative abundance of 19 microorganisms compared with the control sausage. Overall, inoculation with *D. hansenii* SH4 significantly increased the relative abundances of many microorganisms in dry sausage, in particular, strains belonging to the *Leuconostoc*, *Lactobacillus*, and *Debaryomyces* genera, while it decreased the relative abundances of strains belonging to the *Weissella* and *Staphylococcus* genera. The predominant strains, including *L. fallax, L. sakei, L*. *mesenteroides*, and *D. hansenii* (which were abundant in the Dh-SH4 sausage), and *W. jogaejeotgali* and *S. saprophyticus* (which were abundant in the control sausage) showed the most remarkable differences. These strains may serve as typical species to distinguish between the two dry sausages.

### 3.4. Functional Gene Distribution

**Figure 3:**
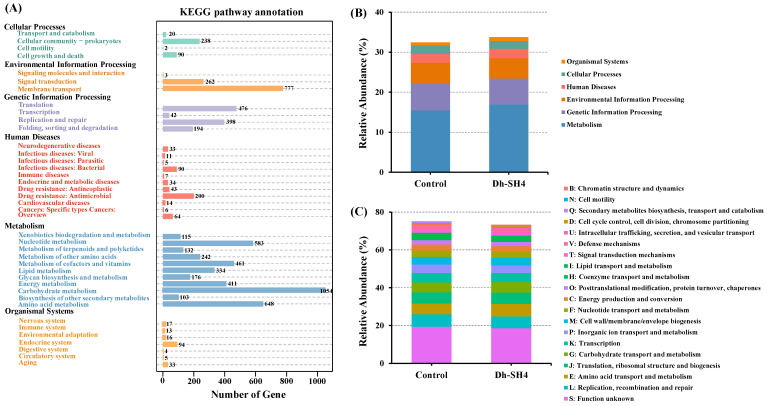
Numbers (**A**) and relative abundances (**B**) of predicted genes in the KEGG pathways, as well as relative abundance (**C**) of predicted genes in the eggNOG pathways (**C**) within the microbial community in the dry sausages. Control: natural fermentation; Dh-SH4: inoculation with *D. hansenii* SH4.

The genes of the control and DH-SH4 sausages were annotated in the KEGG and eggNOG databases to predict the metabolic potential of the microbial communities. The KEGG database facilitates the systematic analysis of gene functions in cellular metabolic pathways and classifies biological metabolic pathways into six categories [25]. As shown in Figure 3A, the KEGG annotation revealed a broad metabolic capacity of the microbiota in the dry sausage, with a predominance of genes annotated to metabolism, followed by those annotated to genetic information processing and environmental information processing. The relative abundances of genes annotated to genetic information processing and environmental information processing were higher in the control sausage than in the Dh-SH4 sausage, whereas the relative abundances of genes annotated to metabolism were higher in the Dh-SH4 sausage (Figure 3B). Further annotation analysis using the KEGG database demonstrated the relationships between these metagenomic genes and several secondary metabolite pathways. The highest number of genes were annotated to carbohydrate metabolism, followed by amino acid metabolism. These results suggest that inoculation with *D. hansenii* SH4 may enhance the microbial activity associated with dry sausage metabolism of carbohydrates and amino acids.

EggNOG is a database for functional annotation of the directly homologous clusters of the constructed genes [26]. As shown in Figure 3C, the functional classes that accounted for a large number of genes in dry sausages were L (replication, recombination, and repair), E (amino acid transport and metabolism), J (translation, ribosome structure, and biogenesis), and G (carbohydrate transport and metabolism). The relative abundances of functional categories L and J were higher in the control sausage than in the DH-SH4 sausage, whereas functional categories G and E were higher in the DH-SH4 sausage. This finding is consistent with the results annotated in the KEGG database, which demonstrated that *D. hansenii* SH4 is favorable for the metabolism of amino acids and carbohydrates in dry sausages.

### 3.5. Biosynthetic Potential of the Microbiota for Producing Volatile Compounds

Carbohydrates, amino acids, and lipids are the primary precursors of flavor compounds in dry sausage, and their metabolism is most directly linked to the development of flavor [2]. Therefore, gene-annotated carbohydrate, amino acid, and lipid metabolic functions were further predicted using the KEGG database.

#### 3.5.1. Carbohydrate Metabolic Pathways

**Figure 4:**
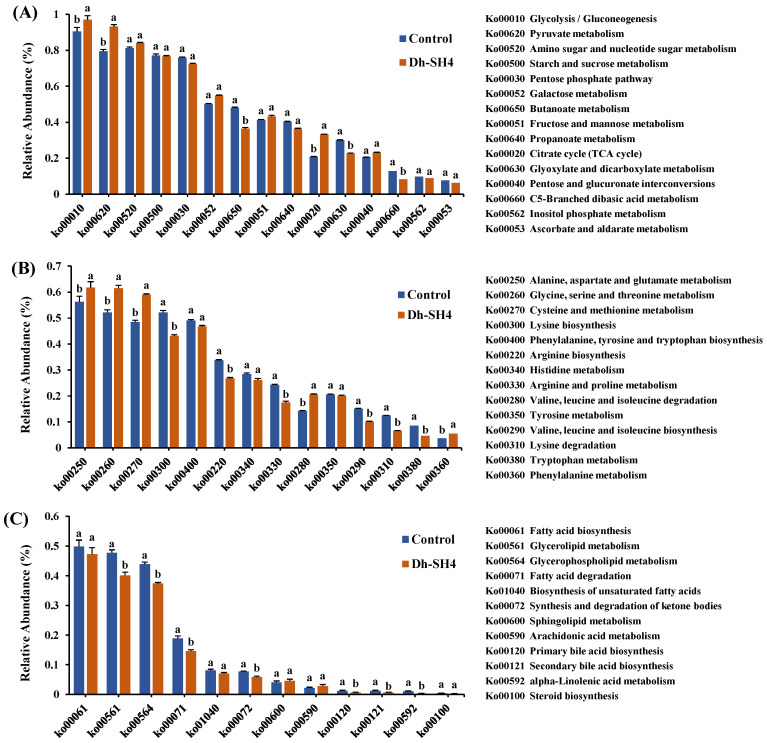
Relative abundance of genes associated with carbohydrate metabolism (**A**), genes associated with amino acid metabolism (**B**), and genes associated with lipid metabolism (**C**) within the microbial community in the dry sausages. Genes were predicted in the KEGG pathways. ^a,b^ Refers to the significant differences between the dry sausages (*p* < 0.05).Control: natural fermentation; Dh-SH4: inoculation with *D. hansenii* SH4.

The relative abundances of genes annotated to the carbohydrate metabolic pathway are shown in Figure 4A. Genes annotated to the Ko00010 (glycolysis/gluconeogenesis) pathway exhibited the highest relative abundance, followed by those annotated to the Ko00620 (pyruvate metabolism) and Ko00520 (amino sugar and nucleotide sugar metabolism) pathways. When compared with the control sausage, the abundances of genes annotated to glycolysis/gluconeogenesis, pyruvate metabolism, and Ko00020 (citric acid cycle) were significantly higher in the Dh-SH4 sausage (*p* < 0.05). These results may explain the high ethanol content found in the Dh-SH4 sausage (Figure 1A) because ethanol is produced mainly through glycolytic and pyruvate metabolism [18]. The main carbohydrate in dry sausage is glucose, which is derived from auxiliary materials added during sausage preparation. Glucose is a vital source of energy for microbial growth. It is involved in the glycolysis pathway of central carbon metabolism and is linked to the synthesis of various metabolites [27]. However, except for the glucose added during sausage preparation, the content of other carbohydrates in the dry sausage was low. Thus, the increased abundance of genes annotated to the gluconeogenic pathway in the Dh-SH4 sausage may indicate the conversion of amino acids to glucose during the fermentation. The key enzymes and coding genes in the formation pathways of volatile compounds were annotated in the KEGG database based on data from metagenomic sequencing. The relative contributions of microorganisms to the formation of volatile compounds were further clarified by calculating the proportion of microorganisms containing the coding genes. The enzymes, genes, and microorganisms involved in the metabolism of carbohydrates to produce volatile compounds such as acetic acid, butanoic acid, ethanol, 2,3-butanediol, and 3-hydroxy-2-butanone are shown in Table 1. The metagenomic results were annotated to the entire glycolytic pathway.

The glycolysis pathway comprises many sequential reactions catalyzed by numerous enzymes, resulting in the breakdown of glucose into pyruvate [7]. After the production of pyruvate, the pathway diverges based on the availability of oxygen [28]. When the fermentation processes occur in an anaerobic environment, pyruvate is converted into lactic acid through lactic acid fermentation, contributing to the distinct sour taste of dry sausages. In an aerobic environment, pyruvate enters the mitochondria and is converted into acetyl-CoA through the enzymatic activity of pyruvate dehydrogenase. Acetyl-CoA is a critical link in multiple metabolic pathways. It not only serves as the essential component for the citric acid cycle but also participates in the synthesis and metabolic pathways of various compounds, such as amino acids and fatty acids [29]. For example, *Bacillus* species may produce acetic acid from acetyl-CoA via the action of acetate kinase (EC 2.7.2.1), while *L. fallax*, *S. saprophyticus*, *L. sakei*, *L. curvatus*, and *L. plantarum* may convert acetyl phosphate into acetic acid via EC 2.7.2.1. Under the catalytic effect of alcohol dehydrogenase (EC 1.1.1.1), alcohol can be used as a substrate to produce acetaldehyde in *L. plantarum*, *L. curvatus*, *L. sakei*, *D. hansenii*, *W. minor*, and the *Candida* species. Acetaldehyde can be further converted into acetic acid by aldehyde dehydrogenase (NAD+) (EC 1.2.1.3). The genes encoding EC 1.2.1.3 were found to be enriched in *Staphylococcus* species.

In the butanoate metabolic pathway, pyruvate can be used as a precursor for producing 3-hydroxy-2-butanone and 2,3-butanediol. *L. sakei*, *L. curvatus*, *L. fallax*, *Weissella paramesenteroides*, *W. jogaejeotgali*, and *Staphylococcus* species may transform pyruvate into 3-hydroxy-2-butanone by the action of acetolactate synthase (EC 2.2.1.6) and acetolactate decarboxylase (EC 4.1.1.5). Subsequently, *L. fallax*, *W. jogaejeotgali*, and *Staphylococcus* species may transform 3-hydroxy-2-butanone into 2,3-butanediol via (R, R)-butanediol dehydrogenase (EC 1.1.1.4) and (S, S)-butanediol dehydrogenase (EC 1.1.1.76). In addition, acetyl-CoA can generate butyrate in the butanoate metabolic pathway by the action of acetyl-CoA acetyltransferase (EC 2.3.1.9), which was mostly associated with *L. curvatus*, *W. minor*, and *L. sakei* in this study. Overall, these results indicated that the most abundant species involved in the volatile compound formation by the carbohydrate metabolic pathway are *L. plantarum*, *L. curvatus*, *L. sakei*, *D. hansenii*, *W. minor*, *L. fallax*, *S. saprophyticus*, *W. paramesenteroides*, and *Candida* species.

#### 3.5.2. Amino Acid Metabolic Pathways

Protein is an important dry sausage component, which is degraded to amino acids through endogenous enzymes in the muscle and microbial enzymes during fermentation [30]. Amino acids are a source of nitrogen for microorganisms and are also precursors of various enzymes and volatile compounds [31]. Figure 4B shows the relative abundances of the genes annotated to amino acid metabolic pathways in the KEGG database. In dry sausages, the genes annotated to the alanine, aspartate, and glutamate metabolism were the most abundant, followed by those annotated to the glycine, serine, and threonine metabolism. Alanine, aspartate, and glutamate can be transformed into each other, and alanine and aspartate can also be catabolized to produce 3-hydroxy-2-butanone [32]. Serine and threonine can be converted to glycine, which can produce pyruvate and, in turn, generate ethanol, acetic acid, and acetyl-CoA, among other compounds [21]. In fermented products, the amino acid precursors of volatile compounds mainly include aromatic amino acids (phenylalanine, tryptophan, and tyrosine), branched-chain amino acids (leucine, isoleucine, and valine), and sulfur-containing amino acids (methionine and cysteine) [31]. The abundances of genes annotated to these pathways were significantly higher in the Dh-SH4 sausage than in the control sausage (*p* < 0.05). Leucine and isoleucine were degraded to branched-chain aldehydes, alcohols, and acids such as 3-methylbutanal, 3-methylbutanol, and 3-methylbutanoic acid in dry sausages [33]. Phenylalanine was metabolized to benzaldehyde, phenylacetaldehyde, acetophenone, benzyl alcohol, and phenethyl alcohol, which further produced aromatic esters such as benzyl acetate, phenethyl acetate, and ethyl 3-phenylpropionate [34].

The enzymes, genes, and microorganisms associated with amino acid metabolism for producing volatile compounds are shown in Table 1. In the valine, leucine, and isoleucine degradation pathways, leucine is first transaminated to its corresponding keto acid by branched-chain amino acid transaminase (EC 2.6.1.42). This keto acid is then converted into 3-methylbutanal, which can be further reduced to 3-methylbutanol or oxidized to 3-methylbutanoic acid by branched-chain keto acid dehydrogenase (EC 1.2.4.4) and other downstream enzymes. The genes encoding EC 2.6.1.42 and EC 1.2.4.4 were found to be enriched in *L. fallax*, *W. minor*, and *Candida* and *Bacilli* species. In the phenylalanine metabolic pathway, phenylalanine is converted to phenylpyruvate by histidinol-phosphate aminotransferase (EC 2.6.1.9), tyrosine aminotransferase (EC 2.6.1.5), and D-alanine transaminase (EC 2.6.1.21), and then converted to phenylacetaldehyde by phenylpyruvate decarboxylase (EC 4.1.1.43). However, only EC 2.6.1.9 and EC 2.6.1.21 were annotated in the dry sausage, and these enzymes were mainly associated with *L. fallax* and the *Bacilli* species. Phenylacetaldehyde can be further converted to phenethyl alcohol by aryl-alcohol dehydrogenase (EC 1.1.1.90), which was mainly associated with *L. curvatus* and *L. sakei* in this study.

#### 3.5.3. Lipid Metabolic Pathways

Lipids are also major sources of volatile compounds [35]. The fatty acids (hexanoic acid, heptanoic acid, octanoic acid, nonanoic acid, and decanoic acid), aldehydes (nonanal and hexanal), alcohols (hexanol, heptanol, 2-heptanol, 2-nonanol, 2-ethylhexanol, 1-octen-3-ol, and trans-2-octen-1-ol) and methyl ketones (2-nonanone) detected in the dry sausages were mainly derived from lipid metabolism. The relative abundances of genes in the lipid metabolic pathways, as annotated by the KEGG database, are shown in Figure 4C. The Ko00061 (fatty acid biosynthesis) pathway had the highest relative abundance of genes, followed by the Ko00561 (glycerolipid metabolism), Ko00564 (glycerophospholipid metabolism), and Ko00071 (fatty acid degradation) pathways. The fatty acids detected in dry sausage can be produced through fatty acid biosynthesis, glycerolipid metabolism, and glycerophospholipid metabolic pathways via the action of microorganisms. In the fatty acid degradation pathway, long-chain fatty acids are degraded into medium- or short-chain fatty acids. Fatty acids can then form aldehydes, ketones, and alcohols such as hexanal, nonanal, hexanol, and heptanol [33], which have been found in dry sausages. Regarding the relative abundances of genes annotated to the fatty acid biosynthesis pathways, no significant differences (*p* > 0.05) were found between the DH-SH4 sausage and the control sausage. However, the relative abundances of the genes annotated to the glycerolipid metabolism, glycerophospholipid metabolism, and fatty acid degradation pathways were significantly (*p* < 0.05) lower in the Dh-SH4 sausage, indicating that the relative abundances of genes related to fatty acid production in dry sausage decreased after inoculation with *D. hansenii* SH4. These findings on the abundance of genes associated with lipid metabolism agree with the content of volatile compounds in dry sausage (i.e., lower contents of fatty acids, aldehydes, ketones, and alcohols derived from lipid metabolism were detected in the Dh-SH4 sausage compared with the control sausage) (Figure 1B).

The enzymes, genes, and microorganisms involved in lipid metabolism for the production of volatile compounds are shown in Table 1. Fatty acids are characteristic flavor compounds in dry sausage and are important precursors of many volatile compounds. In the fatty acid biosynthesis pathway, fatty acid is synthesized de novo with acetyl-CoA as the precursor or by carbon chain extension based on existing fatty acids [36]. Acetyl-CoA can directly enter the fatty acid biosynthesis pathway, or it can first be converted into malonyl-CoA by acetyl-CoA carboxylase (EC 6.4.1.2) before entering the fatty acid synthesis pathway. Subsequently, carbon chain extension and fatty acid release are carried out under the catalytic actions of 3-hydroxyacyl-CoA dehydrogenase (EC 1.1.1.35), medium-chain acyl-[acyl-carrier-protein] hydrolase (EC 3.1.2.21), and long-chain acyl-CoA synthetase (EC 6.2.1.3). Here, the main microorganisms annotated by these enzymes during fatty acid synthesis were *L. fallax*, *L. plantarum*, *L. sakei*, *L. curvatus*, and the *Staphylococcus* species. Fatty acids can also be generated in the glycerolipid metabolic pathway by triacylglycerol lipase (EC 3.1.1.3), and the main microorganisms annotated by this enzyme in this study were *D. hansenii*, *Mycobacterium abscessus*, and the *Staphylococcus* species.

The fatty acid degradation pathway (fatty acid β-oxidation) mainly involves the formation of methyl ketones, secondary alcohols, aldehydes, and fatty acid derivatives. In this pathway, the genes in the microbiota of dry sausage were annotated to EC 1.1.1.35, acetyl-CoA acyltransferase (EC 2.3.1.16), EC 2.3.1.9, EC 1.1.1.1, and aldehyde dehydrogenase (NAD+) (EC 1.2.1.3). These enzymes were mainly associated with *L. plantarum*, *L. curvatus*, *L. sakei*, *D. hansenii*, *W. minor*, and the *Candida* species. Fatty acids can also be automatically oxidized to produce aldehydes (e.g., linoleic acid and linolenic acid oxidation) [37]. Our results suggest that fatty acid peroxygenase (EC 1.11.2.4) may be involved in the auto-oxidation of lipids, but this enzyme is not present in the metabolic pathway and has not been annotated to the relevant microorganisms.

#### 3.5.4. Ester Biosynthetic Pathways

Esters play a crucial role in enhancing the flavor of fermented meat products by providing floral, fruity, and sweet aromas while also contributing to covering any undesirable odors [38]. After fermentation, the most remarkable differences in the volatile profiles were that the types and contents of various esters increased significantly in the Dh-SH4 sausage compared to the control sausage (*p* < 0.05) (Figure 1B). The enzymes, genes, and relative contributions of microorganisms related to ester production are presented in Table 1. Although there is no clear pathway for ester synthesis in the KEGG database, the generation of ester precursors (acids and alcohols) and enzymes may be investigated. Esters are mainly produced by esterification and alcoholysis during fermentation [39]. Esterification is the reaction between an alcohol and an acid to produce esters under the catalysis of an esterase [4]. EC 3.1.1.1 (carboxylate esterase) and EC 3.1.1.3 (lipase) are esterases that not only catalyze the synthesis of esters but can also perform the reversible hydrolysis of esters. The esterification pathway is the most common pathway for the microbial synthesis of esters. Lactic acid bacteria, yeasts, and *Staphylococcus* make important contributions to the esterification process [22]. In our results, *D. hansenii*, *L. curvatus*, *L. sakei*, *M. abscessus*, and *Staphylococcus* species were the main microorganisms associated with esterases in the dry sausage.

Alcoholysis is basically a transferase process in which fatty acyl groups are directly transferred from acyl-CoA derivatives and acylglycerols to alcohols. This reaction is usually catalyzed by an acyltransferase [39]. Our results showed that alcohol O-acetyltransferase (EC 2.3.1.84) was annotated by the microbiota of the dry sausage. This enzyme transfers the acyl groups of acetyl-CoA and fatty acyl-CoA to alcohol to form esters. EC 2.3.1.84 mainly exists in yeast and plays an important role in the synthesis of esters. In this study, EC 2.3.1.84 was found to be enriched in *D. hansenii* and the *Candida* species. EC 1.1.1.1 also plays an important role in the formation of esters, not only reducing aldehydes and ketones to alcohols but also oxidizing hemiacetal compounds to form esters [40].

### 3.6. Metabolic Pathway Networks of Volatile Compounds

**Figure 5:**
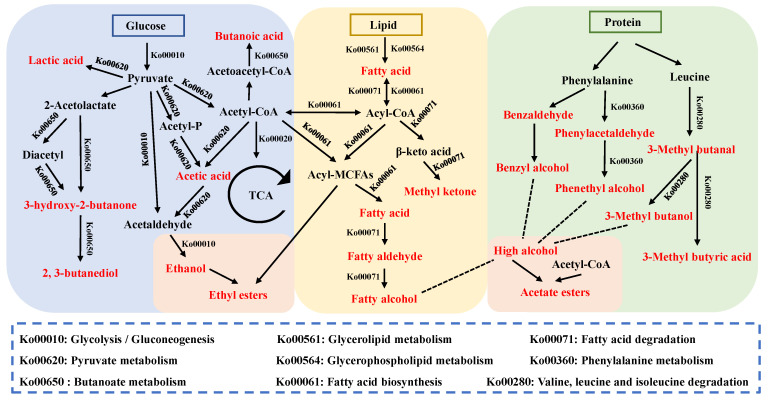
Predicted metabolic pathway network for the volatile compound formation of dry sausages.

The formation of the flavor profile is an intricate process due to the multitude of reactions involved. Figure 5 illustrates the network pathway for the synthesis of volatile compounds, as derived from the KEGG database. In glucose metabolism, glucose generates pyruvate through the glycolysis pathway. Pyruvate is the key node substance for the formation of various volatile compounds and also generates acetaldehyde, acetic acid, ethanol, lactic acid, 3-hydroxy-2-butanone, and 2,3-butanediol through multiple metabolic pathways. In addition, pyruvate through Ko00620 can generate another important key node substance: acetyl-CoA. Acetyl-CoA is a bridge between carbohydrate and lipid metabolism. In lipid metabolism, lipids can be degraded to fatty acids by the combined action of endogenous enzymes and microbial enzymes. In addition, fatty acids can be generated by the fatty acid biosynthesis and fatty acid degradation pathways. In the latter, the corresponding aldehydes, ketones, and alcohols are generated. Endogenous enzymes in the muscle, as well as microbial enzymes, may degrade muscle proteins into amino acids. Compounds derived from phenylalanine and leucine metabolism, including phenylacetaldehyde, phenethyl alcohol, benzaldehyde, benzyl alcohol, 3-methylbutanal, 3-methylbutanol, and 3-methylbutanoic acid, were detected in the dry sausages. Regarding the formation of esters, on the one hand, ethanol from the carbohydrate metabolism reacts with acyl-CoA from the lipid metabolism to synthesize ethyl esters (e.g., ethyl acetate, ethyl butyrate, ethyl caproate, ethyl heptanoate, ethyl octanoate, and ethyl decanoate). On the other hand, higher alcohols from lipid metabolism and amino acid metabolism react with acetyl-CoA to synthesize acetates (e.g., benzyl acetate, phenethyl acetate, and isoamyl acetate).

The functional features of the microbial community were deduced by analyzing the metabolic annotations of functional genes within the KEGG pathways. The results revealed that *Debaryomyces* (especially *D. hansenii*), *Lactobacillus* (mainly *L. curvatus*, *L. sakei*, and *L. plantarum*), *Leuconostoc* (especially *L. fallax*), *Weissella* (especially *W. minor*), and the *Staphylococcus* and *Candida* species were the predominant functional microbes involved in flavor development in dry sausage. In summary, this study revealed the effect of the inoculation with *D. hansenii* SH4 on the structure of the microbial community, as well as the mechanisms behind flavor formation in dry sausage.

## 4. Conclusions

The present study identified significant differences in the microbial diversity and metabolic patterns between the dry sausage inoculated with *D. hansenii* SH4 and naturally fermented sausage. The inoculation with *D. hansenii* SH4 substantially increased the relative abundances of several microorganisms in dry sausage, especially species belonging to the *Leuconostoc*, *Lactobacillus*, and *Debaryomyces genera*, while it decreased the relative abundances of species belonging to the *Weissella* and *Staphylococcus* genera. The *D. hansenii* SH4 inoculation enhanced the microbial activity related to carbohydrate and amino acid metabolism. In addition, diverse species had genes involved in the synthesis of different volatile compounds. *D. hansenii*, *L. curvatus*, *L. sakei*, *L. plantarum*, *L. fallax*, *W. minor*, and the *Staphylococcus* and *Candida* species were the predominant functional microbes identified that involved in flavor development in dry sausage. The flavor formation mechanisms associated with *D. hansenii* SH4 inoculation, as revealed by metagenomic sequencing, are significant because they are valuable for the regulation of the fermentation process. Therefore, this study offers a novel insight into the utilization of microbial resources in dry sausage manufacturing, which may drive the dry sausage industry’s technical progress.

## Figures and Tables

**Table 1:** The enzymes, functional genes, and microorganisms associated with the volatile compound formation in dry sausages.

Volatile Compound	Metabolism Pathway	Enzyme	Enzyme ID	Gene ID	Relative Contributions (%)	
					Control	Dh-SH4
Ethanol	Glycolysis	alcohol dehydrogenase	1.1.1.1	Adh, adhP, YiaY, frmA, adhE	*L. plantarum* (22.29) *L. curvatus* (16.27) *L. sakei* (8.82) *D. hansenii* (7.32) g_*Candida* (6.51)	*L. plantarum* (20.55) *D. hansenii* (15.98) *W. minor* (11.06) g_*Candida* (7.24) *L. curvatus* (5.31)
Acetic acid	Pyruvate metabolism	aldehyde dehydrogenase (NAD+)	1.2.1.3	ALDH	g_Staphylococcus (17.45)	g_*Staphylococcus* (24.09)
		acetate kinase	2.7.2.1	ackA	*L. fallax* (31.15) *L. curvatus* (21.51) *L. plantarum* (7.37)	*L. sakei* (39.30) *L. fallax* (27.97) *L. curvatus* (14.74)
		acylphosphatase	3.6.1.7	acyP	*L. fallax* (70.69) *S. saprophyticus* (9.91)	*L. fallax* (65.18) *L. sakei* (24.94)
		acetyl-CoA synthetase	6.2.1.1	ACSS	c_*Bacilli* (34.15)	c_*Bacilli* (33.11)
butanoic acid	Butanoate metabolism	acetaldehyde dehydrogenase	1.2.1.10	adhE	*L. curvatus* (18.47) *L. sakei* (6.03)	*W. minor* (23.40) *L. sakei* (11.56)
		acetyl-CoA acetyltransferase	2.3.1.9	ACAT, atoB	*L. curvatus* (10.77) *Weissella minor* (6.76)	*L. sakei* (41.53) *L. curvatus* (18.44)
3-Hydroxy-2-butanone 2,3-Butanediol	Butanoate metabolism	(R, R)-butanediol dehydrogenase (R)-acetoin dehydrogenase	1.1.1.4 1.1.1.303	BDH, butB	*L. fallax* (55.49) g_*Staphylococcus* (27.67)	*L. fallax* (84.41) g_*Staphylococcus* (13.30)
		(S, S)-butanediol dehydrogenase (S)-acetoin dehydrogenase	1.1.1.76 1.1.1.304	butA, budC	*L. fallax* (69.60) *W. jogaejeotgali* (9.86)	*L. fallax* (83.59) *W. jogaejeotgali* (1.30)
		acetolactate synthase	2.2.1.6	ilvB, ilvG, ilvI	*L. sakei* (41.61) *L. curvatus* (16.69)	*L. sakei* (50.97) g_*Weissella* (15.64)
		acetolactate decarboxylase	4.1.1.5	alsD, budA, aldC	*L. fallax* (44.07) *W. paramesenteroides* (15.54)	*L. fallax* (74.43) *L. curvatus* (5.59)
Branched-chain compounds derived from amino acid metabolism	Valine, leucine, and isoleucine biosynthesis	branched chain keto acid dehydrogenase	1.2.4.4	BCKDHA, bkdA1	c_*Bacilli* (54.31) g_*Candida* (4.36)	c_*Bacilli* (51.63) g_*Candida* (6.19)
		branched-chain amino acid aminotransferase	2.6.1.42	ilvE	*L. fallax* (51.40) *W. minor* (7.97)	*L. fallax* (79.84) *W. minor* (5.57)
Aromatic compounds derived from amino acid metabolism	Phenylalanine metabolism	aryl-alcohol dehydrogenase	1.1.1.90	E1.1.1.90	*L. curvatus* (61.45) *L. sakei* (8.56) *D. hansenii* (3.15)	*L. sakei* (58.05) *D. hansenii* (17.32) *L. curvatus* (11.95)
		histidinol-phosphate aminotransferase	2.6.1.9	hisC	*L. fallax* (49.82)	*L. fallax* (77.63)
		D-alanine transaminase	2.6.1.21	dat	c_*Bacilli*	c_*Bacilli*
Volatile compounds derived from fatty metabolism	Fatty acid biosynthesis	medium-chain acyl-[acyl-carrier-protein] hydrolase	3.1.2.21	MCH	*L. fallax* (66.55) *L. curvatus* (8.05)	*L. fallax* (68.33) *L. sakei* (23.02)
		long-chain acyl-CoA synthetase	6.2.1.3	ACSL, fadD	g_*Staphylococcus*	g_*Staphylococcus*
		acetyl-CoA carboxylase	6.4.1.2	accC, accD, accA	*L. fallax* (54.21) *L. plantarum* (4.38)	*L. fallax* (79.24) *L. sakei* (4.07)
	Fatty acid degradation	alcohol dehydrogenase	1.1.1.1	Adh, adhP, yiaY, frmA, adhE	*L. plantarum* (22.29) *L. curvatus* (16.27) *L. sakei* (6.82) *D. hansenii* (7.32) g_*Candida* (6.51)	*L. plantarum* (20.55) *D. hansenii* (15.98) *W. minor* (11.06) g_*Candida* (7.24) *L. curvatus* (5.31)
		3-hydroxyacyl-CoA dehydrogenase	1.1.1.35	fadN	k_*Bacteria*	k_*Bacteria*
		aldehyde dehydrogenase (NAD+)	1.2.1.3	ALDH	g_*Staphylococcus* (17.45)	g_*Staphylococcus* (24.09)
		acetyl-CoA acetyltransferase	2.3.1.9	ACAT, atoB	*L. curvatus* (10.77) *W. minor* (6.76)	*L. sakei* (41.53) *L. curvatus* (18.44)
		acetyl-CoA acyltransferase	2.3.1.16	fadA, fadI	c_*Bacilli*	c_*Bacilli*
	—	fatty-acid peroxygenase	1.11.2.4	CYP152A	—	—
	Glycerolipid metabolism	triacylglycerol lipase	3.1.1.3	Lip, TGL2	g_*Staphylococcus* (36.08) *D. hansenii* (17.90) *M. abscessus* (6.31)	g_*Staphylococcus* (37.98) *D. hansenii* (8.80) *M. abscessus* (2.74)
esters	—	carboxylesterase	3.1.1.1	yvaK	*D. hansenii* (8.74) *L. curvatus* (5.22)	*D. hansenii* (23.53) *L. sakei* (3.41)
	Glycerolipid metabolism	triacylglycerol lipase	3.1.1.3	Lip, TGL2	g_*Staphylococcus* (36.08) *D. hansenii* (17.90) *M. abscessus* (6.31)	g_*Staphylococcus* (37.98) *D. hansenii* (8.80) *M. abscessus* (2.74)
	—	alcohol O-acetyltransferase	2.3.1.84	ATF	*D. hansenii* (5.49) *g_Candida* (3.13)	*D. hansenii* (16.04) *g_Candida* (3.75)
	Glycolysis	alcohol dehydrogenase	1.1.1.1	Adh, adhP, yiaY, frmA, adhE	*L. plantarum* (22.29) *L. curvatus* (16.27) *L. sakei* (8.82) *D. hansenii* (7.32) g_*Candida* (6.51)	*L. plantarum* (20.55) *D. hansenii* (15.98) *W. minor* (11.06) g_*Candida* (7.24) *L. curvatus* (5.31)

Control: natural fermentation; Dh-SH4: inoculation with *D. hansenii* SH.

## Data Availability

The original contributions presented in this study are included in the article. Further inquiries can be directed to the corresponding author.

## Appendix A

**Table A1:** Physical properties and microbial counts of naturally fermented sausage and sausage inoculated with *D. hansenii* SH4.

	Control	Dh-SH4
Moisture content (%)	22.83 ± 0.24 ^b^	26.32 ± 0.29 ^a^
*a*_w_	0.752 ± 0.006 ^b^	0.776 ± 0.005 ^a^
pH	5.29 ± 0.05 ^b^	5.53 ± 0.06 ^a^
Yeast count (log CFU/g)	5.62 ± 0.06 ^b^	7.78 ± 0.08 ^a^
LAB count (log CFU/g)	7.36 ± 0.08 ^b^	7.85 ± 0.07 ^a^

^a,b^ Refers to the significant differences between the dry sausages (*p* < 0.05). Control: natural fermentation; Dh-SH4: inoculation with *D. hansenii* SH4.

**Table A2:** The contents (µg/kg) of volatile compounds of naturally fermented sausage and sausage inoculated with *D. hansenii* SH4.

Volatile Compound	CAS	Control	Dh-SH4	Odor Description
**Aldehydes**				
3-Methylbutanal	590-86-3	1.36 ± 0.14	1.49 ± 0.12	chocolate, nutty, cocoa
Hexanal	66-25-1	21.18 ± 0.92 ^a^	16.85 ± 0.89 ^b^	fatty, fresh, green
Nonanal	124-19-6	17.05 ± 0.39 ^a^	10.87 ± 0.22 ^b^	waxy, aldehydic, rose
Benzaldehyde	100-52-7	23.51 ± 0.96 ^a^	11.74 ± 0.35 ^b^	strong, sharp, sweet
Phenylacetaldehyde	122-78-1	2.70 ± 0.15 ^b^	16.91 ± 0.68 ^a^	green, sweet, floral
Cinnamaldehyde	104-55-2	5.28 ± 0.36	5.37 ± 0.25	sweet, spicy, aldehydic
**Ketones**				
3-Hydroxy-2-butanone	513-86-0	50.02 ± 1.86 ^a^	37.51 ± 0.94 ^b^	sweet, buttery, creamy
2-Heptanone	107-87-9	n.d.	n.d.	sweet, fruity, ethereal
2-Nonanone	821-55-6	4.67 ± 0.25 ^a^	2.66 ± 0.32 ^b^	fresh, sweet, green
2,3-Octanedione	585-25-1	n.d.	n.d.	dill, asparagus, cilantro
Acetophenone	98-86-2	n.d.	2.03 ± 0.17	sweet, pungent, hawthorn
Fenchone	1195-79-5	35.18 ± 1.05	37.21 ± 1.39	camphoreous
**Acids**				
Acetic acid	64-19-7	87.01 ± 2.13 ^a^	79.67 ± 2.94 ^b^	sharp, pungent, sour
Butanoic acid	107-92-6	30.32 ± 1.05 ^a^	15.72 ± 0.56 ^b^	sharp, cheese, butter
3-Methylbutanoic acid	503-74-2	5.29 ± 0.42 ^a^	2.37 ± 0.12 ^b^	sour, stinky, cheese
Hexanoic acid	142-62-1	87.72 ± 2.45	92.54 ± 2.42	sour, fatty, sweat
Heptanoic acid	111-14-8	7.94 ± 0.15 ^a^	6.40 ± 0.22 ^b^	rancid, sour, cheesy
Octanoic acid	124-07-2	17.23 ± 0.31 ^a^	2.31 ± 0.26 ^b^	fatty, waxy, rancid
Nonanoic acid	112-05-0	14.46 ± 0.61 ^a^	6.59 ± 0.23 ^b^	waxy, dirty, cheese
Decanoic acid	334-48-5	18.81 ± 0.34 ^a^	6.72 ± 0.29 ^b^	unpleasant, rancid, sour
**Alcohols**				
Ethanol	64-17-5	656.18 ± 5.28 ^b^	938.18 ± 5.86 ^a^	alcoholic, strong, ethereal
2,3-Butanediol	513-85-9	33.01 ± 1.55	36.61 ± 1.61	creamy, fruity, buttery
Pentanol	71-41-0	n.d.	n.d.	fusel, balsam, fermented
3-Methyl-1-butanol	78-83-1	0.64 ± 0.13	0.79 ± 0.04	ethereal, winey, cortex
Hexanol	111-27-3	12.06 ± 0.54 ^a^	10.24 ± 0.43 ^b^	herbal, ethereal, fruity
Heptanol	111-70-6	8.22 ± 0.74 ^a^	6.39 ± 0.65 ^b^	green, musty, leafy
2-Heptanol	543-49-7	2.58 ± 0.14 ^b^	3.49 ± 0.17 ^a^	fresh, lemon, grass
6-Methyl-5-hepten-2-ol	1569-60-4	2.13 ± 0.12 ^a^	1.67 ± 0.05 ^b^	sweet, green, coriander
Octanol	111-87-5	9.05 ± 0.73	8.76 ± 0.57	waxy, green, orange
2-Ethylhexanol	104-76-7	6.69 ± 0.13	7.58 ± 0.65	citrus, fresh, floral
1-Octen-3-ol	3391-86-4	9.51 ± 0.86 ^a^	6.73 ± 0.64 ^b^	mushroom, earthy, green
trans-2-Octen-1-ol	18409-17-1	16.74 ± 0.43 ^a^	7.38 ± 0.41 ^b^	fatty, green, citrus
2-Nonanol	628-99-9	7.19 ± 0.38	n.d.	waxy, green, creamy
Benzyl alcohol	100-51-6	n.d.	9.57 ± 0.63	floral, rose, phenolic
Phenethyl alcohol	60-12-8	3.09 ± 0.25 ^b^	14.64 ± 0.97 ^a^	floral, rose, dried
Cineole	470-82-6	250.37 ± 3.38	244.41 ± 2.63	herbal, eucalyptus, camphor
Linalool	78-70-6	231.47 ± 3.02	238.92 ± 3.35	citrus, floral, sweet
α-Terpineol	98-55-5	20.04 ± 0.49	21.43 ± 0.27	terpenic, pine, lilac
Terpinen-4-ol	562-74-3	66.87 ± 1.77	74.72 ± 2.83	pepper, woody, musty
Nerolidol	40716-66-3	20.50 ± 0.92	21.59 ± 0.58	floral, green, citrus
**Esters**				
Ethyl acetate	141-78-6	105.16 ± 2.13 ^b^	150.33 ± 2.41 ^a^	ethereal, fruity, sweet
Ethyl lactate	97-64-3	50.25 ± 2.64 ^b^	62.49 ± 2.76 ^a^	fruity, sharp, tart
Methyl butyrate	623-42-7	10.51 ± 0.59 ^b^	13.10 ± 0.88 ^a^	fruity, apple, sweet
Ethyl butyrate	105-54-4	125.02 ± 3.09 ^b^	150.70 ± 2.18 ^a^	fruity, pineapple, cognac
Ethyl 2-methylbutyrate	7452-79-1	n.d.	5.43 ± 0.13	fruity, apple berry
Ethyl valerate	539-82-2	15.16 ± 0.46 ^b^	26.05 ± 1.24 ^a^	fruity, sweet, apple
Ethyl isovalerate	108-64-5	11.00 ± 0.21 ^b^	18.70 ± 0.98 ^a^	fruity, sweet, apple
Isoamyl acetate	123-92-2	n.d.	3.24 ± 0.19	sweet, fruity, banana
Methyl hexanoate	106-70-7	121.06 ± 2.53 ^b^	194.78 ± 2.72 ^a^	fruity, pineapple, ether
Ethyl hexanoate	123-66-0	685.73 ± 7.94 ^b^	791.54 ± 7.47 ^a^	fruity, sweet, pineapple
Ethyl heptanoate	106-30-9	15.57 ± 0.43 ^b^	21.34 ± 0.18 ^a^	fruity, pineapple, cognac
Ethyl caprylate	106-32-1	18.28 ± 0.81 ^b^	25.58 ± 1.73 ^a^	fruity, wine, waxy
Ethyl caprate	110-38-3	n.d.	16.77 ± 0.26	waxy, sweet, fruity
Benzyl acetate	140-11-4	n.d.	1.27 ± 0.09	floral, jasmin, fresh
Phenethyl acetate	103-45-7	n.d.	7.24 ± 0.48	floral, rose, honey
Ethyl 3-hydroxybutyrate	5405-41-4	n.d.	2.33 ± 0.17	fruity, green, grape
Ethyl 3-hydroxyhexanoate	2305-25-1	n.d.	n.d.	fruity, grape, cranberry
Ethyl 3-phenylpropionate	2021-28-5	n.d.	12.19 ± 0.31	hyacinth, rose, honey
Bornyl acetate	76-49-3	21.54 ± 0.37	22.10 ± 1.03	balsamic, woody, pine
Geranyl acetate	105-87-3	18.46 ± 0.41	17.85 ± 0.28	floral, rose, lavender
Linalyl acetate	115-95-7	5.51 ± 0.17	6.40 ± 0.38	herbal, sweet, green
Ethyl laurate	106-33-2	n.d.	13.53 ± 0.85	waxy, floral, soapy
Methyl salicylate	119-36-8	n.d.	17.32 ± 0.53	wintergreen, mint, sweet
**Alkenes**				
Sabinene	3387-41-5	44.55 ± 1.26	41.20 ± 1.39	woody, terpenic, citrus
Myrcene	123-35-3	41.91 ± 1.07	38.57 ± 1.59	spicy, peppery, terpene
Dipentene	5989-27-5	782.18 ± 4.20	779.29 ± 5.09	citrus, orange, fresh
γ-Terpinene	99-85-4	111.56 ± 3.29	111.75 ± 2.25	terpenic, oily, woody
α-Copaene	3856-25-5	10.89 ± 0.76	11.28 ± 0.47	woody, spicy, honey
α-Curcumene	644-30-4	15.68 ± 0.86	16.66 ± 0.32	herbal, fresh, woody
β-Caryophyllene	87-44-5	144.38 ± 2.70	138.36 ± 3.09	spicy, sweet, woody
Terpinolene	586-62-9	21.14 ± 0.46	21.10 ± 0.78	herbal, fresh, woody
p-Cymene	99-87-6	87.08 ± 2.18	82.93 ± 2.92	terpenic, fresh, citrus
α-Pinene	7785-70-8	12.29 ± 0.38	10.58 ± 0.65	herbal, terpenic, aromatic
β-Pinene	127-91-3	22.44 ± 1.17	20.50 ± 1.06	woody, resinous, pine
3-Carene	13466-78-9	13.47 ± 0.62	11.90 ± 0.49	citrus, terpenic, herbal
α-Phellandrene	99-83-2	22.39 ± 1.28	22.12 ± 1.30	citrus, herbal, lime
β-Elemene	515-13-9	6.73 ± 0.30	6.28 ± 0.43	sweet
α-Caryophyllene	6753-98-6	16.41 ± 0.68	15.97 ± 0.57	woody
**Others**				
D-Camphor	464-49-3	39.16 ± 1.25	42.18 ± 1.53	camphoreous, minty phenolic
4-Allylanisole	140-67-0	350.49 ± 5.32	346.13 ± 4.40	anisic, sweet, spicy
L-Borneol	464-45-9	11.00 ± 0.61	12.07 ± 0.40	pine, woody, camphor
Anethol	104-46-1	512.03 ± 4.32	514.18 ± 4.29	sweet, anisic, licorice
Safrole	94-59-7	38.50 ± 1.34	40.98 ± 2.01	sweet, warm, spicy
Eugenol	97-53-0	304.92 ± 4.34	303.11 ± 3.97	spicy, sweet, clove
Methyl eugenol	93-15-2	8.15 ± 0.49	7.67 ± 0.63	sweet, fresh, warm

n.d.: not detected. ^a,b^ Refers to the significant differences between the dry sausages (*p* < 0.05). Control: natural fermentation; Dh-SH4: inoculation with *D. hansenii* SH4. The bold in the table refers to the classification of volatile compounds.

**Table A3:** Statistical information of the metagenomic sequence data and bioinformatics analysis.

	Control-1	Control-2	Control-3	Dh-SH4-1	Dh-SH4-2	Dh-SH4-3
Raw data (Mbp)	6213.48	6663.19	6132.97	6537.33	6687.53	6663.51
Clean data (Mbp)	6166.05	6636.61	6101.13	6512.53	6665.27	6641.50
Clean Q20 (%)	97.73	97.68	95.05	97.63	97.56	97.50
Clean Q30 (%)	94.18	93.95	90.17	93.92	93.78	93.63
Clean GC (%)	45.01	42.85	45.11	44.01	44.08	44.01
Scaftigs number	54,579	59,624	56,031	61,846	64,733	65,129
Scaftigs average length (bp)	1437.31	1465.77	1374.02	1201.80	1266.19	1548.90
Scaftigs N50 length (bp)	1896	1997	1716	1386	1534	2476
ORFs number	36,475	40,912	35,795	37,949	39,155	40,490
Unigenes number	61,921	62,991	61,108	64,118	64,385	64,368

Control: natural fermentation; Dh-SH4: inoculation with *D. hansenii* SH4.
